# Exploring Neurocognitive and Emotional Outcomes of Long COVID: A Study Among Pakistani Patients

**DOI:** 10.7759/cureus.67815

**Published:** 2024-08-26

**Authors:** Muddsar Hameed, Mahrukh Anwar Abbasi, Fatima Noor, Ayesha Fatima, Muhammad Ibrahim, Shah Bano, Ali Hamza, Ali Afaq Rasool Malik, Muhammad Ahsan Saeed, Saman Iqbal

**Affiliations:** 1 Department of Neuroscience, Brain Tech Clinic and Research Center, Islamabad, PAK; 2 Department of Internal Medicine, Foundation University Medical College, Islamabad, PAK; 3 Department of Medicine, Sheikh Zayed Medical College, Rahim Yar Khan, PAK; 4 Department of Medicine, Shifa International Hospital, Islamabad, PAK; 5 Department of Medicine, King Edward Medical University, Lahore, PAK

**Keywords:** mental health, cognitive function, emotional outcomes, neurocognitive outcomes, long covid

## Abstract

Background and objective

Coronavirus disease 2019 (COVID-19), primarily a respiratory illness, also significantly impacts neurocognitive and emotional health, particularly in its long-term manifestation known as long COVID. This study aimed to investigate the neurocognitive and emotional outcomes of long-term COVID-19 in Pakistani patients, to address the persisting symptoms and their effects on mental health and cognitive function.

Methods

A cross-sectional study involving 100 adult participants who had been COVID-19-free was conducted in Islamabad between March 2022 and March 2023. Participants were assessed using the Mini-Mental State Examination (MMSE), attention-deficit/hyperactivity disorder (ADHD) Self-Report Questionnaire, Satisfaction with Life Scale (SWLS), and Punishing Allah Reappraisal Scale. Data were analyzed using SPSS Statistics v26 (IBM Corp., Armonk, NY), employing chi-square tests, t-tests, and ANOVA.

Results

The study revealed significant correlations between COVID-19 symptoms and psychological variables. COVID-19 symptoms showed a negative correlation with MMSE scores (r = -0.04, p<0.01) and positive correlations with ADHD (r = 0.13, p<0.05), depression (r = 0.14, p<0.05), and anxiety (r = 0.25, p<0.05). Females reported higher levels of depression [mean: 1.21, standard deviation (SD): 0.83] and anxiety (mean: 1.33, SD: 0.86) compared to males.

Conclusions

Our findings highlight the extensive impact of long-term COVID-19 on neurocognitive and emotional health, with significant gender differences observed in emotional outcomes. These results emphasize the need for integrated mental health services in post-COVID-19 care plans, as well as gender-sensitive interventions.

## Introduction

While coronavirus disease 2019 (COVID-19) is predominantly a respiratory infection, it may also affect other organs, including the brain [1]. Exhausting fatigue, sadness, and neurocognitive problems like brain fog, physical discomfort, and weakness are common symptoms of long COVID, which lasts longer than 3-12 weeks after severe acute respiratory syndrome coronavirus 2 (SARS-CoV-2) infection [2]. Myalgia (10.9%), exhaustion (5.5%), breathlessness (6.1%), cough (2.1%), sleeplessness (1.4%), mood disorders (0.48%), and anxiety (0.6%) are the most prevalent symptoms, according to Naik et al. [3].

Post-COVID-19 syndrome is defined as a state that follows COVID-19 in individuals with a possible or confirmed history of infection. It typically manifests three months after the commencement of COVID-19 symptoms and lasts for at least two months and cannot be attributed to any other diagnosis [4]. Several recent studies have documented immunological abnormalities, including increased activation of the innate immune system, myalgia/chronic fatigue syndrome, encephalomyelitis, fibromyalgia, depression, and other mental health disorders [5]. SARS-CoV-2, which causes severe acute respiratory syndrome, is responsible for this condition. To cause severe neurological dysfunction or to interfere with peripheral nerves and cause sensory loss, skeletal muscle discomfort, disorientation, diminished consciousness, acute cerebrovascular disease, and epilepsy, SARS-CoV-2 may attack the central nervous system.

Moreover, neuropsychiatric diseases can manifest as encephalopathy, mood swings, suicidal thoughts, sleeplessness, psychosis, and cognitive impairment [6]. The specific psychophysiology processes of COVID-19 and the underlying etiology of its neurological, cognitive, and psychiatric dysfunctions are presumably complicated, and they have not yet been determined [7]. Concerns have been raised over the COVID-19 pandemic's potential impact on mental health as well as its impending threat to public health worldwide. People with severe mental illness are especially vulnerable to global health emergencies such as the COVID-19 pandemic [8].

China initially notified the nation's WHO Country Office on December 31, 2019, regarding the emergence of an unidentified kind of pneumonia in Wuhan, the provincial capital of Hubei. The disease quickly spread from China to at least 20 other nations. As a result, on January 30, 2020, the Director General of WHO declared COVID-19 a Public Health Emergency of International Concern. The IHR 2005 regulations (https://www.who.int/ihr/publications/9789241580496/en/) have defined what a PHIEC is. The WHO did not give this pandemic the name COVID-19 until February 11, 2020. On February 25, 2020, the first COVID-19 case was identified in Pakistan; on March 29, 2020, the first death due to the condition was announced. Pakistan reported 14 fatalities and 1,597 cases in the 67th WHO report, which was released on March 29 [9].

Measures taken to stop the spread of COVID-19, public health campaigns, and transportation constraints that make it difficult to obtain medication or seek medical attention quickly may make people more likely to experience psychotic episodes or worsen the symptoms of pre-existing psychotic illnesses [10]. This pattern is in line with what we see in those who experience sadness, anxiety, and addiction [11]. One important clinical aspect of the illness with potential long-term consequences is post-COVID-19 cognitive decline [12]. A COVID-19 infection can impair mental functioning, leading to deficits in areas such as language learning, working memory, set-shifting, divided attention, motor speed, and executive processes [13,14].

## Materials and methods

We examined the neurocognitive and affective effects of long COVID in Pakistani patients via a quantitative, cross-sectional study conducted in Islamabad. The study included adult participants (aged 18 years and older) who gave their informed consent. The inclusion criteria were as follows: participants who had a confirmed diagnosis of COVID-19, those who had recovered from the acute phase of the illness at least three months before the study, and those who had reported experiencing persistent symptoms consistent with long COVID. Additionally, patients with a history of pre-existing neurocognitive or severe psychiatric disorders before their COVID-19 diagnosis were excluded, as these could confound the study's results. Data collection was conducted from March 2022 to March 2023 since, by the end of 2022, the number of COVID-19 cases in Pakistan had considerably decreased. Data collection started in January 2022 when the ethical approval for the study was received. Data were collected from tertiary care hospitals in Islamabad.

Sample and sampling technique

The study comprised individuals over the age of 18 years who gave their informed written consent. However, those who previously used psychiatric medicine or had serious cognitive or psychiatric problems were not allowed to participate in the study. Our estimated sample size was 100 using the WHO sample size calculator with a 95% confidence interval (CI) and an expected population proportion. Due to time and resource constraints, we used a non-probability convenience sampling strategy to gather data from 100 individuals.

To assess the neurocognitive and emotional outcomes of long COVID in Pakistani patients, we employed several validated instruments. The Mini-Mental State Examination (MMSE), developed by Dr. Marshall Folstein, Dr. Susan Folstein, and Dr. Paul McHugh in 1975, was used to evaluate participants' neurocognitive functions. This widely used cognitive assessment tool evaluates various cognitive functions, including orientation, registration, attention and calculation, recall, language, and visuospatial skills. The maximum score is 30 points, with scores below 24 generally indicative of cognitive impairment. The MMSE has been validated against other cognitive measures and is widely accepted in clinical and research settings as a reliable indicator of cognitive impairment (see Appendices).

To assess symptoms of ADHD, we used the ADHD Self-Report Questionnaire, a 15-item self-report measure designed to capture the presence and severity of ADHD symptoms as experienced by the participants (see Appendices). The Satisfaction with Life Scale (SWLS), developed by Ed Diener, Robert A. Emmons, Randy J. Larsen, and Sharon Griffin in 1985, was employed to measure participants' global cognitive judgments of life satisfaction. This scale consists of five items, each rated on a 7-point Likert scale ranging from 1 (strongly disagree) to 7 (strongly agree) (see Appendices).

Additionally, we used two self-report questionnaires to assess depression and anxiety symptoms, providing a comprehensive overview of participants' emotional well-being and mental health status. To assess the religious coping mechanism, we used the Punishing Allah Reappraisal Scale, a psychological measure that explores individuals' perceptions of divine punishment in response to life problems, reflecting their religious coping strategies. This scale includes items that delve into beliefs about being punished by Allah for past actions, wondering about the reasons for divine punishment, and feeling punished for a lack of devotion (see Appendices). These tools enabled us to comprehensively assess the neurocognitive and emotional outcomes of long COVID in the study population.

We developed a questionnaire to collect data, which included demographic-related questions, information about COVID-19, the MMSE, the ADHD scale, the SWLS scale, and the Punishing Allah Reappraisal scale. Participants were asked to provide their responses based on their current condition and their condition a few weeks prior. All participants included in this study had suffered from COVID-19. Their consent was obtained before completing the questionnaire. Before starting the study, ethical approval had been obtained from the Brain Tech Clinic Research Center's Institutional Review Board and Ethical Committee (BTC-IRB-2022-0011). The purpose of the study, the voluntary nature of participants' involvement, and their right to withdraw at any moment before, during, or after the study were explained to the participants. The participants' privacy, identity, and sense of self-respect were preserved at all times.

The data were analyzed using SPSS Statistics v26 (IBM Corp., Armonk, NY). Both analytical and descriptive statistics, such as chi-square tests, independent sample t-tests, ANOVA, and mean and standard deviation computations, were used to analyze the data. A cutoff point for significance of p<0.05 was utilized.

## Results

Table 1 shows the intercorrelation relationships between the study variables, indicating significant associations at different levels. COVID symptoms showed a significant negative correlation with MMSE scores (r = -0.04) and a positive correlation with ADHD (r = 0.13), punishment (r = 0.03), depression (r = 0.14), and anxiety (r = 0.25). This suggests that as the severity of COVID symptoms increases, cognitive function decreases slightly, while ADHD symptoms, feelings of punishment, depression, and anxiety increase.

**Table 1:** Intercorrelation relationship between study variables ^*^P<0.05. ^**^P<0.01 (statistically significant) P-values were calculated using Spearman's correlation, with the significance levels set at p<0.05 and p<0.01 ADHD: attention-deficit/hyperactivity disorder; COVID-19: coronavirus disease 2019; MMSE: Mini-Mental State Examination

Variables	1	2	3	4	5	6	7
1. COVID-19 symptoms	-	-	-	-	-	-	-
2. MMSE	-0.04^**^	-	-	-	-	-	-
3. ADHD	0.13^*^	-0.07	-	-	-	-	-
4. Punishment	0.03^*^	-0.31	-0.12	-	-	-	-
5. Life satisfaction	-0.10^**^	0.01	0.28^**^	-0.21^*^	-	-	-
6. Depression	0.14^*^	-0.13	-0.47^**^	0.07	-0.17	-	-
7. Anxiety	0.25^*^	-0.08	-0.42^**^	0.15	-0.24^*^	0.51^**^	-

MMSE scores, indicating cognitive function, were negatively associated with life satisfaction (r = -0.10), meaning that lower cognitive function is linked to lower life satisfaction. ADHD was positively correlated with life satisfaction (r = 0.28) but negatively with depression (r = -0.47) and anxiety (r = -0.42), indicating that higher ADHD symptoms were associated with higher life satisfaction but lower depression and anxiety. Punishment was negatively correlated with life satisfaction (r = -0.21), suggesting that feelings of punishment reduce life satisfaction. Life satisfaction was negatively correlated with depression (r = -0.17) and anxiety (r = -0.24), indicating that higher life satisfaction is associated with lower levels of depression and anxiety. Depression and anxiety were positively correlated (r = 0.51), showing that these two mental health issues often occur together. These findings highlight the complex interplay between cognitive function, mental health, and life satisfaction, emphasizing the importance of addressing multiple dimensions of well-being in affected individuals.

Table 2 displays the distribution of cognitive deficits across various demographic variables including gender, COVID-19 symptoms, educational level, and age, with chi-square (χ2) values and corresponding p-values indicating statistical significance. In terms of gender, males constituted 34% (n = 34) of the sample, with 22 having normal cognition, four experiencing mild cognitive deficits, four moderate deficits, and four severe deficits. Females made up 66% (n = 66) of the sample, with 28 showing normal cognition, 16 mild deficits, 12 moderate deficits, and 10 severe deficits.

**Table 2:** Distribution of cognitive deficits by gender, COVID-19 symptoms, education, and age ^*^Significance level set at p<0.05 and p<0.01 COVID-19: coronavirus disease 2019

Variables		N (%)	Normal cognition	Mild cognitive deficit	Moderate cognitive deficit	Severe cognitive deficit	χ^2^	P-value^*^
Gender	Male	34 (34)	22	4	4	4	4.7	0.01
	Female	66 (66)	28	16	12	10		
COVID-19 symptoms	Mild	42 (42)	24	6	7	5	3.3	0.04
	Moderate	16 (16)	17	11	7	7		
	Severe	16 (16)	9	3	2	2		
Education	Matric	11 (11)	6	3	0	2	4.4	0.05
	Intermediate	12 (12)	8	1	2	1		
	Graduation	77 (77)	36	14	16	11		
Age	Young adults	77 (77)	33	15	15	10	9.8	0.02
	Middle-aged adults	22 (22)	14	5	0	3		
	Old adults	3 (3)	1	0	1	1		
	Senior old adults	2 (2)	2	0	0	0		

The chi-square value of 4.7 with a p-value of 0.01 indicates a statistically significant association between gender and cognitive deficits. Regarding COVID-19 symptoms,42 (42%) participants had mild symptoms, 16 (16%) moderate, and 16 (16%) severe symptoms. Among those with mild symptoms, 24 had normal cognition, six mild deficits, seven moderate deficits, and five severe deficits. Among participants with moderate symptoms, 17 had normal cognition, 11 had mild deficits, seven had moderate deficits, and seven had severe deficits. Severe symptoms were associated with nine normal, three mild, two moderate, and two severe deficits. The chi-square value of 3.3 with a p-value of 0.04 suggests a significant relationship between the severity of COVID-19 symptoms and cognitive deficits.

In terms of educational level, 11 (11%) had a matric-level education, 12 (12%) intermediate, and 77 (77%) graduated. Among those with matric-level education, six had normal cognition, three had mild deficits, two had severe deficits, and none had moderate deficits. Among those with intermediate-level education, eight had normal cognition, one mild deficit, two moderate deficits, and one severe deficit. Among graduates, 36 had normal cognition, 14 mild deficits, 16 moderate deficits, and 11 severe deficits. The chi-square value of 4.4 with a p-value of 0.05 indicates a significant association between educational level and cognitive deficits.

Age distribution shows that 77 (77%) were young adults, 22 (22%) middle-aged adults, three (3%) old adults, and two (2%) were senior old adults. Among young adults, 33 had normal cognition, 15 mild deficits, 15 moderate deficits, and 10 severe deficits. The middle-aged adult group had 14 with normal cognition, five with mild deficits, none with moderate deficits, and three with severe deficits. The old adult group had one with normal cognition, none with mild deficits, one with moderate deficits, and one with severe deficits. Among senior adults, two had normal cognition, and none had cognitive deficits. The chi-square value of 9.8 with a p-value of 0.02 shows a significant relationship between age and cognitive deficits. Overall, the data indicate significant associations between gender, COVID-19 symptoms, education level, and age with the distribution of cognitive deficits, with p-values lower than the thresholds of 0.05 and 0.01.

Table 3 presents a comparison of psychological variables between male and female participants. Females had significantly higher levels of depression and anxiety compared to males, with Cohen's d values of -0.54 (p = 0.01) and -0.69 (p = 0.04) respectively, indicating medium to large effect sizes. In terms of mental state, males showed slightly better scores than females, with a small effect size (Cohen's d = 0.27, p = 0.02). Males also scored higher on ADHD measures, with a moderate effect size (Cohen's d = 0.43, p = 0.00). Life satisfaction scores were similar between males and females, with no significant difference (Cohen's d = 0.03, p = 0.10). Finally, for the Punishing Allah Reappraisal variable, males scored higher than females, showing a small to moderate effect size (Cohen's d = 0.37, p = 1.1). These findings suggest that there are notable gender differences in several psychological variables, with females experiencing higher levels of depression and anxiety, while males exhibit higher ADHD scores and slightly better mental states. Life satisfaction appears to be comparable between genders.

**Table 3:** Comparison between genders regarding psychological variables ^*^P-values were calculated using independent sample t-tests, with significance level set at p<0.05 ADHD: attention-deficit/hyperactivity disorder; CI: confidence interval; LL: lower limit; SD: standard deviation; UL: upper limit

Comparison between genders									
Variable	Male		Female				95% CI		
	Mean	SD	Mean	SD	t (98)	P-value^*^	LL	UL	Cohen's D
Depression	0.73	0.89	1.21	0.83	2.6	0.01	-0.83	-1.1	-0.54
Anxiety	0.70	0.90	1.33	0.86	3.3	0.04	-0.99	-0.25	-0.69
Mental state	22.27	7.95	20.03	8.26	1.2	0.02	-1.1	5.2	0.27
ADHD	23.73	5.13	21.68	4.15	2.1	0.00	0.16	3.5	0.43
Life satisfaction	16.11	4.15	15.96	3.49	0.1	0.10	-1.1	1.7	0.03
Punishing Allah Reappraisal	7.94	3.24	6.71	3.48	1.7	1.1	-0.18	2.6	0.37

Table 4 shows a comparison of psychological variables across different COVID-19 severity levels, revealing notable differences. Depression showed no significant differences, with an F value of 1.1 and an effect size (η²) of 0.02. Anxiety scores showed a trend towards higher severity, resulting in an F value of 3.1 and an effect size of 0.06. Mental state scores were significantly different across severity levels, reflected by an F value of 0.98 and an effect size of 0.02 (p<0.05). ADHD scores did not show significant variation, with an F value of 0.94 and an effect size of 0.01. Life satisfaction also showed significant differences, having an F value of 0.84 and an effect size of 0.01 (p<0.05). Lastly, Punishing Allah Reappraisal showed no significant differences, with an F value of 0.12 and an effect size of 0.04.

**Table 4:** Comparison of psychological variables across different COVID-19 severity levels ^*^P<0.05 considered significant (calculated by ANOVA) ADHD: attention-deficit/hyperactivity disorder; COVID-19: coronavirus disease 2019; SD: standard deviation; η²: effect size

Variables	Mild COVID-19	Moderate COVID-19	Severe COVID-19	F (2,97)	η²
-	Mean ±SD	Mean ±SD	Mean ±SD	-	-
Depression	0.9 ±0.8	1.0 ±0.8	1.3 ±0.8	1.1	0.02
Anxiety	0.8 ±0.8	1.2 ±0.9	1.5 ±0.8	3.1	0.06
Mental state	21.6 ±8.4	19.4 ±8.1	22.1 ±7.6	0.98^*^	0.02
ADHD	23.0 ±4.4	22.0 ±4.9	21.5 ±3.8	0.94	0.01
Life satisfaction	15.5 ±3.6	16.2 ±3.3	16.8 ±4.6	0.84^*^	0.01
Punishing Allah Reappraisal	7.3 ±3.4	6.9 ±3.5	7.0 ±3.2	0.12	0.04

## Discussion

The exploration of neurocognitive and emotional outcomes of long-term COVID-19 is crucial due to the extensive and lingering impact of COVID-19 on individuals' health and well-being. Long COVID remains a relatively new and poorly understood condition, and this study contributes valuable data on its neurocognitive and emotional effects, aiding in the development of targeted interventions and therapies. From a public health perspective, by highlighting the prevalence of neurocognitive and emotional symptoms in Pakistani patients, the study underscores the need for robust public health strategies to support affected individuals, emphasizing the importance of mental health services as part of the post-COVID-19 care plan.

Additionally, the findings can inform policymakers about the necessity of integrating mental health and cognitive rehabilitation services into the healthcare system to address the long-term impacts of COVID-19, leading to more comprehensive and holistic healthcare policies. For clinicians, understanding the specific neurocognitive and emotional challenges faced by long-term COVID-19 patients can improve patient care, allowing for better diagnosis, management, and support for these patients, ultimately enhancing their quality of life. This study also adds to the growing body of literature on long COVID, providing a foundation for future research and identifying areas where further investigation is needed, such as the underlying mechanisms of cognitive and emotional dysfunctions.

The rationale for conducting this study is rooted in the need to address the multifaceted impacts of COVID-19, particularly in the context of Pakistani patients. Pakistan, like many other countries, has faced significant challenges due to the COVID-19 pandemic, with a high number of infections amidst limited healthcare resources. Understanding the long-term impacts of the virus is essential for effective healthcare planning and resource allocation. Mental health is often an overlooked aspect of post-COVID-19 recovery, and this study brings attention to the psychological and cognitive repercussions, advocating for a more integrated approach to patient care that includes mental health services. The sociocultural context of Pakistan can influence the manifestation and reporting of neurocognitive and emotional symptoms. This study provides insights specific to the Pakistani population, which may differ from findings in other regions, thereby enhancing the cultural relevance of the research. Furthermore, the persistent symptoms of long COVID can place additional strain on an already burdened healthcare system.

By identifying the specific needs of long COVID patients, this study aims to support healthcare providers in delivering more effective and targeted care. Understanding the breadth of Long-term COVID symptoms, including their cognitive and emotional aspects, is vital for promoting holistic recovery. This study advocates for comprehensive care strategies that address both physical and mental health, ensuring a more complete recovery process for patients. By investigating the neurocognitive and emotional outcomes of long-term COVID-19 in Pakistani patients, this study aims to fill critical gaps in knowledge, support the development of better healthcare policies, and improve clinical practices to enhance patient outcomes.

Our findings elucidate the neurocognitive and emotional impacts of long-term COVID-19 on Pakistani patients, contributing to a growing body of literature exploring the multifaceted outcomes of COVID-19. Our results reveal significant associations between COVID-19 symptoms and various psychological and cognitive variables, underscoring the extensive and lingering effects of the virus beyond the acute phase of infection. Our study demonstrated a significant negative correlation between COVID-19 symptoms and MMSE scores (r = -0.04, p<0.01), indicating that increased severity of COVID-19 symptoms is associated with reduced cognitive functioning. This aligns with previous research indicating that SARS-CoV-2 can target the central nervous system, leading to cognitive deficits such as memory loss, mental fog, and executive dysfunction [15]. These findings are corroborated by studies showing that post-COVID-19 cognitive decline includes impairments in working memory, attention, and executive functions, which are critical for daily functioning and overall quality of life [16].

The study found significant correlations between COVID-19 symptoms and emotional variables such as depression (r = 0.14, p<0.05) and anxiety (r = 0.25, p<0.05), indicating that the psychological burden of long-term COVID-19 is substantial. These results are consistent with existing literature highlighting the increased prevalence of depression and anxiety among COVID-19 survivors [17]. The heightened levels of depression and anxiety among female participants, as evidenced by higher mean scores compared to males, suggest a gender disparity in the emotional impacts of COVID-19. This finding is significant as it highlights the need for gender-sensitive interventions in post-COVID-19 care [18].

The study revealed a complex and somewhat paradoxical relationship between COVID-19 symptoms, ADHD, and life satisfaction. Interestingly, higher ADHD scores were associated with increased life satisfaction (r = 0.28, p<0.01) but lower depression (r = -0.47, p<0.01) and anxiety (r = -0.42, p<0.01). This unexpected finding may suggest that individuals with ADHD possess unique resilience factors or alternative coping strategies that enable them to maintain higher levels of life satisfaction despite the psychological challenges of COVID-19. For example, previous research has suggested that some individuals with ADHD may employ adaptive coping mechanisms such as cognitive reframing, seeking novel and stimulating experiences, or leveraging hyperfocus in specific activities to buffer against negative emotions [19]. However, these strategies might not be universally protective, as ADHD is also associated with a range of challenges, including difficulties in sustained attention and impulse control, which could impact long-term well-being. Therefore, it is crucial for future studies to further explore these mechanisms in different contexts and populations to better understand the factors that contribute to this paradoxical association.

The significant negative correlation between cognitive function (MMSE scores) and life satisfaction (r = -0.10, p<0.01) underscores the adverse impact of cognitive impairments on overall life satisfaction, emphasizing the necessity of cognitive rehabilitation as part of the recovery process [20]. The association between the Punishment Allah Reappraisal scale and various psychological variables provides insight into the role of religious coping in the Pakistani context. The significant negative correlation between feelings of divine punishment and life satisfaction (r = -0.21, p<0.05) suggests that individuals perceiving their illness as divine punishment experience lower life satisfaction. This highlights the cultural and religious dimensions of coping strategies and their psychological impacts, necessitating culturally sensitive psychological support for long-term COVID-19 patients [21].

Our findings underscore the importance of integrating mental health services into post-COVID-19 care plans. Given the significant neurocognitive and emotional challenges identified, there is a critical need for comprehensive care strategies that address both physical and mental health. This includes cognitive rehabilitation, psychological support, and gender-sensitive interventions to cater to the specific needs of female patients who are disproportionately affected by emotional distress [22]. Moreover, the study emphasizes the importance of public health strategies that prioritize mental health services as part of the broader healthcare response to the pandemic [23].

Future research should focus on longitudinal studies to explore the long-term neurocognitive and emotional outcomes of COVID-19, including the underlying mechanisms of these dysfunctions. Additionally, research should aim to develop and evaluate targeted interventions to mitigate the adverse impacts of long-term COVID-19, particularly in resource-constrained settings like Pakistan. Further studies are also needed to explore the gender differences in psychological impacts and the role of cultural and religious coping mechanisms in the recovery process [24]. Overall, our study highlights the significant neurocognitive and emotional challenges faced by long COVID patients in Pakistan. The findings emphasize the need for comprehensive care strategies that address both physical and mental health, ensuring a more complete recovery process for patients. By providing insights specific to the Pakistani population, this study contributes valuable data to the global understanding of long COVID and recommends the development of more effective and targeted interventions.

Limitations and strengths

While our study provides valuable insights, it is important to acknowledge the limitations inherent in its cross-sectional design. This design restricts our ability to draw conclusions about the directionality or causality of the observed relationships between long-term COVID-19 symptoms and neurocognitive or emotional outcomes. Without temporal data, we cannot determine whether the psychological and cognitive impairments observed are a direct consequence of COVID-19 or if they preexisted and were exacerbated by the virus. Future longitudinal studies are essential to establish causality and to track the progression of these impairments over time. Such studies could also explore potential moderating factors, such as preexisting mental health conditions, socioeconomic status, and access to healthcare, which may influence the trajectory of recovery and the effectiveness of interventions.

Furthermore, the non-probability convenience sampling technique employed in this study may have introduced selection bias, limiting the generalizability of the findings to the broader population. The reliance on self-report measures also introduces the possibility of response bias, where participants may underreport or overreport their symptoms. Finally, the exclusion of individuals with severe psychiatric issues may have led to an underestimation of the true prevalence of neurocognitive and emotional impacts among long-term COVID-19 patients.

However, despite these limitations, our study contributes valuable data on the significant neurocognitive and emotional challenges faced by long-term COVID-19 patients in Pakistan. By providing insights specific to the Pakistani population, this study supports the development of more effective and targeted interventions, ultimately contributing to a more comprehensive understanding of long-term COVID-19 on a global scale.

## Conclusions

Our findings emphasize the need for comprehensive care strategies that integrate both physical and mental health services, addressing the specific needs of affected individuals to ensure a more holistic recovery process. By highlighting these aspects in the Pakistani context, this study contributes to the global understanding of long COVID and supports the development of targeted interventions to mitigate its long-term effects. Furthermore, the significant gender differences observed in emotional impacts call for gender-sensitive approaches in post-COVID-19 care. The integration of mental health services into routine post-COVID-19 healthcare is essential to address the widespread emotional and cognitive repercussions.

Policymakers should consider these findings to develop robust healthcare policies that incorporate cognitive rehabilitation and psychological support as standard practices. Overall, our study underscores the importance of addressing the multifaceted impacts of long COVID to improve patient outcomes and enhance the quality of life for those affected by this persistent condition. Future research should continue to explore the long-term neurocognitive and emotional consequences of COVID-19 and the effectiveness of various intervention strategies to support recovery.

## Appendices

**Table 5:** Mini-Mental State Exam (MMSE)

Section	Task	Points
Orientation	What is the (year) (season) (date) (day) (month)?	5
-	Where are we (state) (country) (town) (hospital) (floor)?	5
Registration	Name 3 objects: 1 second to say each. Then ask the patient all 3 after you have said them. Give 1 point for each correct answer. Then repeat them until he/she learns all 3. Count trials and record. Trials	3
Attention and Calculation	Serial 7's. 1 point for each correct answer. Stop after 5 answers. Alternatively, spell "world" backward	5
Recall	Ask for the 3 objects repeated above. Give 1 point for each correct answer	3
Language	Name a pencil and watch	2
-	Repeat the following “No ifs and buts"	1
-	Follow a 3-stage command: “Take a paper in your hand fold it in half and put it on the floor”	3
-	Read and obey the following: CLOSE YOUR EYES	1
-	Write a sentence	1
-	Copy the design shown	1
Total	-	30

**Table 6:** ADHD rating scale ADHD: attention-deficit/hyperactivity disorder

Items	Response
I have trouble getting started or initiating tasks	-
I start tasks with enthusiasm but lose interest quickly	-
I find it hard to do things that aren't necessary or highly stimulating	-
I am easily distracted by things I see or hear	-
I become absorbed in things or tasks that interest me sometimes to the point of forgetting about people around me or other obligations	-
I have trouble following conversations because I am distracted or because I am trying to remember what I wanted to say	-
I forget things, even when they are important to me	-
At least once a day I lose or misplace items, for example, keys, wallet, purse, or cell phone	-
I consistently forget appointments and, when I do remember, I often am late	-
I can’t seem to get a handle on clutter. My personal space is messy and has piles of papers and miscellaneous items	-
I have difficulty figuring out what is most important or what I should start with given a list of things to do	-
I waste time trying to decide what to do first	-
I become frustrated when things don’t go as planned and can quickly become angry. I often let go of my anger as quickly as it came	-
I have trouble completing multiple-step tasks and moving from one task to Another	-
I say “I will do it later” and then forget all about it	-

**Table 7:** Satisfaction with life scale and psychological well-being items

Items	Responses
In most ways, my life is close to my ideal	-
The conditions of my life are excellent	-
I am satisfied with my life	-
So far I have gotten the important things I want in life	-
If I could live my life over, I would change almost nothing	-
Psychological wellbeing	
Do you experience symptoms of depression, such as persistent sadness, loss of interest, or feelings of hopelessness?	-
Have you experienced increased anxiety or worry since the onset of Long COVID symptoms?	-

**Table 8:** Punishing Allah Reappraisal scale

Items	Responses
When I face a problem in life, I believe I am being punished by Allah for the bad actions I did	-
I face a problem in life; I wonder what I did for Allah to punish me	-
When I face a problem in life, I feel punished by Allah for my lack of devotion	-
